# Lanthanides as Calcium Mimetic Species in Calcium-Signaling/Buffering Proteins: The Effect of Lanthanide Type on the Ca^2+^/Ln^3+^ Competition

**DOI:** 10.3390/ijms24076297

**Published:** 2023-03-27

**Authors:** Valya Nikolova, Nikoleta Kircheva, Stefan Dobrev, Silvia Angelova, Todor Dudev

**Affiliations:** 1 Faculty of Chemistry and Pharmacy, Sofia University “St. Kliment Ohridski”, 1164 Sofia, Bulgaria; 2 Institute of Optical Materials and Technologies “Acad. J. Malinowski”, Bulgarian Academy of Sciences, 1113 Sofia, Bulgaria

**Keywords:** calcium-signaling protein, calcium-buffering protein, lanthanide, calcium mimetic species

## Abstract

Lanthanides, the 14 4f-block elements plus Lanthanum, have been extensively used to study the structure and biochemical properties of metalloproteins. The characteristics of lanthanides within the lanthanide series are similar, but not identical. The present research offers a systematic investigation of the ability of the entire Ln^3+^ series to substitute for Ca^2+^ in biological systems. A well-calibrated DFT/PCM protocol is employed in studying the factors that control the metal selectivity in biological systems by modeling typical calcium signaling/buffering binding sites and elucidating the thermodynamic outcome of the competition between the “alien” La^3+^/Ln^3+^ and “native” Ca^2+^, and La^3+^ − Ln^3+^ within the lanthanide series. The calculations performed reveal that the major determinant of the Ca^2+^/Ln^3+^ selectivity in calcium proteins is the net charge of the calcium binding pocket; the more negative the charge, the higher the competitiveness of the trivalent Ln^3+^ with respect to its Ca^2+^ contender. Solvent exposure of the binding site also influences the process; buried active centers with net charge of −4 or −3 are characterized by higher Ln^3+^ over Ca^2+^ selectivity, whereas it is the opposite for sites with overall charge of −1. Within the series, the competition between La^3+^ and its fellow lanthanides is determined by the balance between two competing effects: electronic (favoring heavier lanthanides) and solvation (generally favoring the lighter lanthanides).

## 1. Introduction

**Figure 1:**
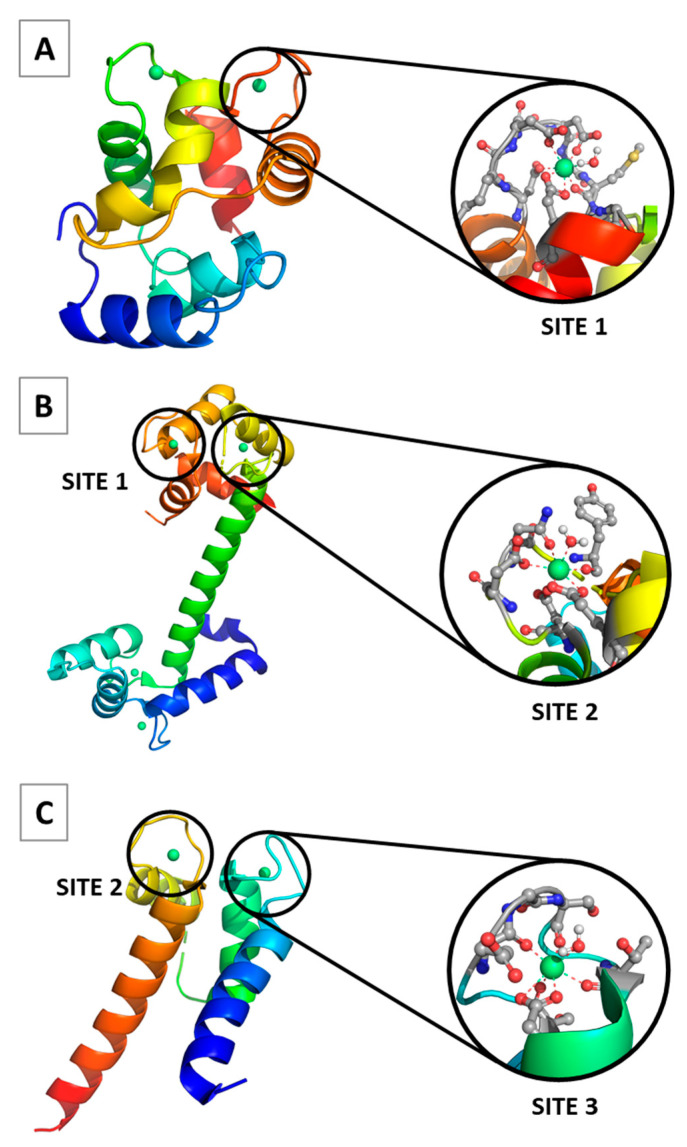
Structures of calcium proteins as given in the protein data bank: (**A**) parvalbumin—PDB entry 1PAL [22]; (**B**) calmodulin D—PDB entry 4DJC [23]; (**C**) S100P—PDB entry 1J55 [24]. Details about Site 1, 2, 3 are given in the text.

Lanthanum (La^3+^) and its fellow lanthanides (Ln^3+^) have been extensively used to study the structure and biochemical properties of vast number of metalloproteins, the calcium-signaling/buffering proteins in particular [1,2,3,4,5,6,7,8,9,10,11,12,13,14,15,16,17,18,19]. The latter (parvalbumin, calmodulin D, calcineurin, recoverin, troponin C, cadherin, and S100P) are involved in a plethora of physiological processes such as muscle contraction, vision, cell cycle regulation, brain cortex and cerebellum modulation, and microtubule organization [20]. These proteins contain Ca^2+^ binding sites, most of which belong to the so-called EF-hand motif family. The canonical EF hand motif, which is highly selective for Ca^2+^ over Mg^2+^ and other physiological metal cations, consists of a 12-residue calcium-binding loop surrounded by two helices creating a signature helix–loop–helix motif. Aspartate/glutamate (Asp/Glu) and asparagine/glutamine (Asn/Gln) side chains as well as backbone carbonyls from the loop ligate the Ca^2+^ ion, which often retains a bound water molecule [21]. The binding site is characterized by a pentagonal bipyramidal geometry. Notably, the conserved Glu at the last position of the EF-hand binding loop (Glu-12) binds Ca^2+^ in a bidentate fashion via both carboxylate oxygens, whereas the other Asp/Glu residues bind Ca^2+^ monodentately, employing one of the carboxylate oxygens (Figure 1).

Employing Ln^3+^ (this includes La^3+^ from now on unless specified) in probing Ca^2+^ binding sites, which have limited number of chemical properties to be examined by experimental techniques, stems from the ability of the lanthanides to closely mimic some characteristics of the native metal. Lanthanides and Ca^2+^ behave as “hard” acids with high affinity to “hard” bases containing oxygen, rather than “soft” bases comprising nitrogen, phosphorus, and sulfur [25]. Ln^3+^ also resembles more alkaline earth metal dications than the respective transition metal counterparts, as the bonding with the former is essentially ionic [26]. Directed “covalent” bonding typically observed in the transition metal compounds is not observed in Ln^3+^ series, mostly because the f-electrons (buried deep in the atom electronic shell) do not play an essential role in the lanthanide–ligand bonding [25,27]. A slight amount of covalency in lanthanide bonds has been attributed to the participation of the lanthanide 6s orbitals rather than the 4f orbitals [28]. Furthermore, lanthanides, exhibiting a range of ionic radii encompassing that of Ca^2+^, appear to be almost ideal biomimetic agents for Ca^2+^. For example, La^3+^ and Ca^2+^ have similar ionic radii: 1.16/1.1 and 1.12/1.06 Å, respectively, for eight/seven-coordinated ions, whereas the respective ones for the lanthanide series (coordination number of eight) are in the range of 1.14 (for the “early” lanthanides such as Ce^3+^) and 0.98 Å (for the “late” lanthanides such as Lu^3+^) [29]. Unlike many other 3+ metal cations (such as Ga^3+^, Al^3+^, and Fe^3+^), Ln^3+^ do not ionize the bound water molecules (the pK_a_ of the aqua ligands in the La–aqua cluster is 9.0 [30]) which resembles the behavior of the divalent metals (Ca^2+^ in particular) which also do not deprotonate the coordinated waters (the pK_a_ for Ca^2+^–aqua complexes is 12.8 [30]).

Triply charged lanthanide cations, however, also possess properties distinct from those of Ca^2+^. Ln^3+^ exhibits higher affinity to the partner ligands than Ca^2+^. Its hydration free energy is also much higher compared to that of Ca^2+^ (see Methods section). The typical coordination number of the two cations in protein binding sites differs as well: it is eight for lanthanides, but seven for Ca^2+^ (especially in EF hand motifs) [31,32]. These differences reflect on their binding properties, as demonstrated below.

Most of the physicochemical characteristics (excluding spectroscopic behavior) of lanthanides within the lanthanide series are similar, and therefore the metals are very often referred to as a single chemical element, Ln, with properties reminiscent of that of the namesake La. Notably, although close, the chemical characteristics of the lanthanides are not identical. For example, the atomic/ionic radii decrease as the atomic number in the series increases (lanthanide contraction). Consequently, their hydration free energy and ligand affinity increase in the same direction. However, no systematic study on the binding affinity/selectivity of the members of the entire lanthanide series, pertinent to the metals’ biochemistry, has been performed (to the best of our knowledge). Thus, several questions remain unaddressed. It is not clear which factors control the metal selectivity within the lanthanide series and which lanthanides are the strongest/poorest Ca^2+^ competitors in biological systems. How do the lanthanides compare with lanthanum itself in substituting for Ca^2+^ in the respective metal binding centers? Could lanthanum be safely employed as a fully-fledged representative of its fellow lanthanides in biochemical reactions? 

In this article, we endeavor to address these questions by modeling typical calcium signaling/buffering binding sites and elucidating the thermodynamic outcome of the competition between “alien” La^3+^/Ln^3+^ and “native” Ca^2+^, and La^3+^—Ln^3+^ within the lanthanide series. A well-calibrated DFT/PCM protocol is employed. A combination between density functional theory (DFT) calculations and polarizable continuum method (PCM) computations is employed in the task. Notably, this approach, unlike other theoretical methods, is very well suited for properly treating interactions in highly charged systems, such as the present ones, between polycationic species and polyanionic clusters. The strong electrostatic interactions between metal cations and protein residues are treated by DFT methods to account explicitly for all electronic effects accompanying the process, including charge transfer to/from the dication/trication and polarization of the participating entities. These calculations can also efficiently treat weaker hydrogen bonding and/or van der Waals interactions between protein and/or water ligands. Furthermore, the reduced size of the system enables the use of sufficiently high-level DFT methods and large basis sets in computing the metal exchange’s free energies for the respective metal binding sites, which would be computationally prohibitive, employing other computational protocols. Since the strength of the metal–ligand interactions rapidly attenuates with increasing distance, the effect of the protein matrix on the Ca^2+^/La^3+^ selectivity is accounted for by employing PCM evaluations with an effective dielectric constant ε, ranging from 4 to 29 to reflect the increasing solvent accessibility of the metal-binding site. It should be noted that our aim here is to obtain reliable trends in the free energy changes with varying parameters of the system such as the composition, overall charge, rigidity and the solvent exposure of the metal binding site, rather than to reproduce the absolute ion exchange free energies in these metal centers. Notably, trends in the free energies computed using this approach have been found to be consistent with experimental observations in previous works [33,34,35,36,37,38,39,40,41,42,43]. The effect of a number of factors on the process (such as the metal’s affinity for the binding site, its ionic radius and hydration energy, the overall charge of the binding center, and the dielectric properties of the medium) is assessed. Note that the trends of changes in the respective thermodynamic quantities with the varying type and characteristics of the respective influencers are evaluated (which is the power of the current computational protocol), rather than reproducing the absolute value of the binding affinity/selectivity of the metal cation.

The competition between rival cations, such as Ca^2+^ and Ln^3+^, in the various model binding sites was evaluated by treating the interactions between the ion and ligands lining the binding pore explicitly using density functional theory (DFT); the region inside the binding site was represented by an effective dielectric constant ε varying from 4 to 29, in order to mimic binding cavities of increasing solvent exposure which are treated by polarizable continuum model (PCM) computations. The binding site selectivity can be expressed in terms of the free energy ΔG_x_ for replacing the Ca^2+^ bound inside the binding cavity with the “alien” Ln^3+^:

[Ln^3+^-aq] + [Ca^2+^-protein] + H_2_O → [Ln^3+^-protein-H_2_O] + [Ca^2+^-aq] (1)

In Equation (1), [Ca^2+^/Ln^3+^-protein] and [Ca^2+^/Ln^3+^-aq] represent the metal cation bound inside the binding pocket and unbound outside the binding cavity (in the bulk solvent), respectively. Note that the metal in the Ca^2+^ binding site is 7-coordinated, whereas in its La^3+^ counterpart lanthanum, it is 8-coordinated, at the expense of an additional first-shell water molecule. A positive free energy for Equation (1) implies a Ca^2+^-selective site, whereas a negative value suggests a Ln^3+^-selective one.

## 2. Results and Discussion

**Figure 2:**
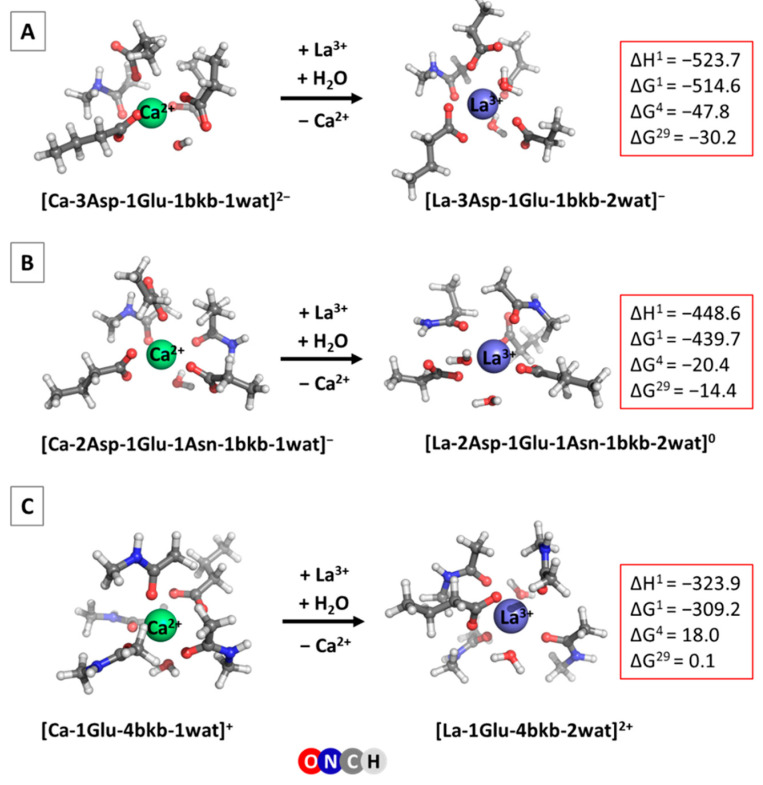
M062X/6-311++G**//SDD fully optimized Ca^2+^ and La^3+^-loaded metal binding sites of calcium-signaling/buffering proteins: (**A**) Site 1, (**B**) Site 2 and (**C**) Site 3. Enthalpies and Gibbs energies (in kcal mol^−1^) of the Ca^2+^ → La^3+^ substitution are also given. Superscript 1 stands for the process in the gas phase, whereas superscripts of 4 and 29 signify metal exchange reactions taking place in buried and solvent-accessible binding pockets, respectively.

Several calcium-binding sites characteristic of Ca^2+^-signaling/buffering proteins were modeled, and their metal selectivity was examined. These are designated as Site 1, Site 2 and Site 3, whose optimized Ca^2+^/La^3+^-loaded structures, following the experimental observations (Figure 1), are presented in Figure 2.

### 2.1. Site 1

This is a canonical EF-hand motif binding site comprising three Asp- and one Glu- side chains, and a peptide backbone group donated by the host protein. Such calcium-selective binding sites (with respect to other “native” cytosolic cations) are typical of parvalbimun (EF site) and calmodulin (EF-I and EF-IV sites). One/two water molecules complement the first coordination shell of the metal cation (Figure 2A). Glutamate is bidentately bound to the Ca^2+^/La^3+^ cations, whereas the aspartates are coordinated to the metal in a monodentate fashion. The overall charge of the metal-free binding pocket is −4. 

Structurally, the two calcium/lanthanum-loaded binding sites differ mostly in their shape and metal coordination number (CN), as Ca^2+^ cation prefers heptacoordination, whereas lanthanum favors higher coordination numbers in biological systems, CN = 8 in particular [21,22]. The metal–ligand bond distances are not, however, very different; the mean of Ca^2+^-O (ligand) distance is 2.43 Å, while the respective La^3+^-O (ligand) distance is 2.55 Å. 

The enthalpies and Gibbs free energies for the Ca^2+^ → La^3+^ metal exchange (Equation (1)) are also presented in Figure 2A. The gas-phase thermodynamics, as seen, are enthalpy-driven, since ΔH^1^ is the major contributor to the free energy of the reaction. As expected, ΔG^1^ is quite negative (−515.6 kcal mol^−1^), signifying very favorable substitution of the dicationic Ca^2+^ by the tricationuc La^3+^ in the gas phase. The solvation, however, strongly attenuates the gas-phase free energy as the desolvation penalty of the incoming La^3+^ species (−751.7 kcal mol^−1^), and strongly outweigh the free energy gain by the outgoing Ca^2+^ (−359.7 kcal mol^−1^) [44]. Nevertheless, ΔGs in the condensed phase remain negative, implying a calcium-binding site vulnerable to the La^3+^ substitution. Buried binding sites (ε = 4) are more predisposed to lanthanum attack (ΔG^4^ = −47.8 kcal mol^−1^) than their solvent-exposed counterparts (ΔG^29^ = −30.2 kcal mol^−1^). The trends outlined above for the Ca^2+^ → La^3+^ substitution are followed by those for the Ca^2+^ → Ln^3+^ exchange as well, where the gas-phase free energies vary between −524.6 and −613.0 kcal mol^−1^, and the ΔG^4^/ΔG^29^ fluctuate between −41.6 and −50.7/−24.0 and −33.3 kcal mol^−1^, respectively (Table 1).

**Figure 3:**
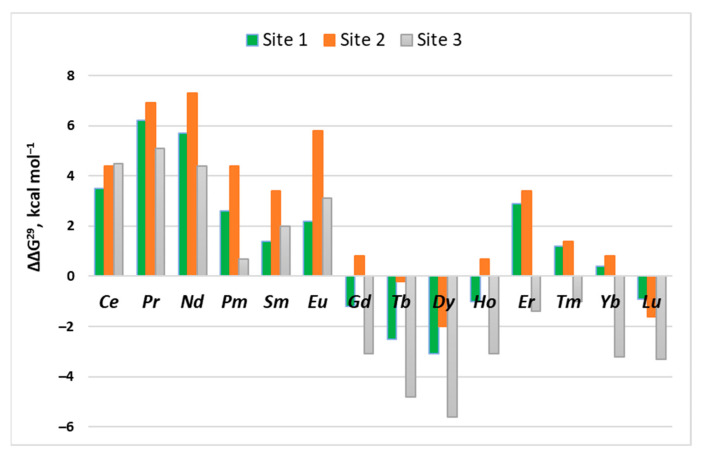
Plot of ΔΔG^29^ (in kcal mol^−1^) for La^3+^ → Ln^3+^ exchange in the series of lanthanides in Site 1, Site 2 and Site 3.

Tracking the changes in the free energies of metal exchange within the lanthanide series is of particular interest. The following reaction was considered:

[Ln^3+^-aq] + [La^3+^-protein] → [Ln^3+^-protein] + [La^3+^-aq] (2)

and the respective La^3+^/Ln^3+^ relative free energies, ΔΔGs, were evaluated (Table 1). The gas-phase ΔΔG steadily decreases going down the series (from −10.0 for Ce^3+^ to −98.4 kcal mol^−1^ for Lu^3+^) as the charge density increases in the same direction (due to decreasing ionic radius; see Section 3.2, Table 4), thus securing more favorable interactions with the host binding side for lanthanides rather than lanthanum. The free energies of solvation of lanthanides, however, also decrease in the same direction (incurring higher desolvation penalties) and strongly affect the outcome of the La^3+^ → Ln^3+^ competition in condensed media. This apparently depends on the balance between the electronic and solvation effects. As can be seen in the second half of Table 2 and the green columns of Figure 3, ΔΔGs do not follow linear dependency but fluctuate between positive and negative values, with the lowest (most favorable) being for Dy^3+^ (ΔΔG^4^/ΔΔG^29^ = −2.9/−3.1 kcal mol^−1^) and the highest (least favorable) for Pr^3+^ (ΔΔG^4^/ΔΔG^29^ = 6.2/6.2 kcal mol^−1^). Interestingly, ΔΔGs distribution seems to follow a semi-sinusoidal pattern (Figure 3; green columns) with a maximum (Pr^3+^), followed by a minimum (Dy^3+^) followed by another (less pronounced) maximum (Er^3+^). 

### 2.2. Site 2

This is a typical EF-hand calcium binding site found in calmodulin (EF-II and EF-III), recoverin (EF-II and EF-III) and S100P (EF-II). The hepta-coordinated Ca^2+^ complex includes two Asp- (monodentately bound to the metal), one Glu- (bidentately bound), one Asn, a backbone peptide group and a water molecule. The La^3+^ cation binds to the same set of ligands, with the addition of another water molecule complementing its coordination number to 8. The optimized structures of the Ca^2+^ and La^3+^ constructs are presented in Figure 2B. The metal-free binding site bears a negative charge of −3. 

The thermodynamic characteristics of the Ca^2+^ → La^3+^ metal exchange are also given in Figure 2B. It can be seen that, as in the above case (Site 1), the reaction is enthalpically driven. The metal substitution appears, again, to be highly favorable in both the gas phase and condensed media. However, the Gibbs free energies are higher (less negative) than the respective quantities in Site 1 (Figure 2A), which implies decreased competitiveness of La^3+^ in Site 2 compared with that in Site 1. This is mainly due to the decreased negative charge density in Site 2 (−3) as compared to that in Site 1 (−4), which affects (unfavorably) the La^3+^-ligand interactions to a greater extent than the respective Ca^2+^-ligand interactions.

The Gibbs free energies for the Ca^2+^ → Ln^3+^ exchange in the lanthanide series are summarized in the first half of Table 2. These fluctuate in certain limits but remain on a negative ground for the entire series, implying that all the lanthanides are effective Ca^2+^ competitors in this type of binding site. Furthermore, examining ΔΔG^4^/^29^ for the La^3+^ → Ln^3+^ substitution, one can conclude that very few of the lanthanides, such as Tb^3+^, Dy^3+^ and Lu^3+^, can successfully compete with La^3+^ for Site 2 (second half of Table 2 and Figure 3, orange columns), as evidenced by negative values for the three cations but positive values for all the other lanthanides. The positive values for the respective lanthanides are the highest in the series of the three binding sites examined here (Figure 3 and Table 1, Table 2 and Table 3).

### 2.3. Site 3

Site 3 represents the calcium binding site found in S100P protein (EF-I). It has a low negative charge density, comprising one bidentate anionic Glu-, four neutral backbone peptide groups and one or two water molecules, depending on the metal. The structure of the optimized Ca^2+^ and La^3+^ complexes and thermodynamic parameters for the metal competition in this system are shown in Figure 2C. 

As expected, in view of the low negative charge density of Site 3 (the overall charge of the metal free binding pocket is −1), the gas-phase free energy gain upon Ca^2+^ → La^3+^ substitution decreases further in absolute value as compared with those in Site 1 and Site 2. This weighs heavily on the condensed-phase ΔG^4/29^, which now are positive, implying calcium binding sites resistant to lanthanum attack.

The free energies of Ca^2+^ → Ln^3+^ exchange in the buried binding sites (ε = 4) remain positive throughout the entire lanthanide series as well (Table 3, left-hand side), but change sign in solvent-accessible binding pockets (ε = 29) for metals from the second half of the series (heavier lanthanides). The heavier lanthanides are also better competitors of the namesake La^3+^ in solvent-exposed binding sites characterized by negative ΔΔG^4/29^ (Table 3, second half and Figure 3, gray columns). Again, Dy^3+^ appears to be the lanthanide with the highest affinity for the protein-binding site.

## 3. Materials and Methods

### 3.1. Models Used

Model Ca^2+^ binding sites were built in accordance with the existing X-ray structures of the respective signaling/buffering proteins deposited in the PDB: 1PAL [22] (parvalbumin), 4DJC [23] (calmodulin), 4YI8 [45] (recoverin) and 1J55 [24] (S100P). The side chains of Asp−, Glu− and Asn were represented by CH_3_CH_2_COO^−^, CH_3_CH_2_CH_2_COO^−^ and CH_3_CH_2_CONH_2_, respectively, whereas the metal-coordinated backbone peptide group was modeled by N-methylacetamide (CH_3_CONHCH_3_). The initial model seven-coordinated Ca^2+^-bound constructs were subjected to geometry optimization (see below). Consequently, Ca^2+^ from the optimized structures was replaced by a La^3+^/Ln^3+^ cation, followed by a water molecule addition (to complement the lanthanide coordination number to 8), and the resulting constructs were fully optimized. The optimized structures of Ca^2+^ and La^3+^ complexes are shown in Figure 2. Notably, the overall structure of the respective Ln^3+^ complexes does not differ significantly from that of the La^3+^ counterpart, and thus these are not depicted here. An oxidation state of 3+, typical of the lanthanum and lanthanides, was considered throughout the paper.

### 3.2. DFT/PCM Calculations

The M062X method [46] in combination with Pople’s triple zeta 6-311++G(d,p) basis set augmented with polarization and diffuse functions for C, H, N, O and Ca atoms, and an SDD basis set/effective core potential for Ln^3+^ (see Table 4) was employed in the calculations. This combination of theoretical method/basis set has been meticulously calibrated/validated in several of our previous studies with respect to available experimental data, and has proven to be dependable, as it reliably reproduced the structure of a series of representative metal constructs [47] as well as the Gibbs free energies of metal substitution in acetate, imidazole and glycine complexes [48]. The M062X/6-311++G(d,p)//SDD level of theory has been recently used in the study of the competition between Ag^+^ and Ni^2+^ in nickel enzymes, and between Ag^+^ and the key constituents of the bacterial cell wall/membrane [49,50].

Each metal-bound structure was optimized in the gas phase by employing a Gaussian 09 suite of programs [51]. Electronic energies, E_el_, were evaluated for each optimized construct. Consequent frequency evaluations were conducted at the same M062X/6-311++G(d,p)//SDD level of theory to confirm a local minimum on the potential energy surface; no imaginary frequency was detected for any of the structures considered. The frequencies were scaled by an empirical factor of 0.983 [46] and used to calculate the thermal energies, E_th_, comprising zero-point energy and entropies, S. The differences ΔE_el_, ΔE_th_ and ΔS between the products and reactants in Equation (1) were used to evaluate the metal substitution Gibbs free energy in the gas phase, ΔG^1^, at T= 298.15 K, according to:

ΔG^1^ = ΔE_el_^1^ + ΔE_th_^1^ − TΔS^1^ (3)

The basis set superposition error for this type of reactions has been found to be insignificant [52], and thus was not taken into account in the present calculations. 

The condensed-phase evaluations were conducted in solvents mimicking the dielectric properties of buried and solvent-accessible binding sites, diethyl ether (ε = 4) and propanonitrile (ε = 29), respectively. Solvation effects were simulated by performing PCM computations employing the solvation model based on density (SMD) scheme [31,53] as implemented in the Gaussian 09 program. In so doing, the optimized gas-phase structure of each metal complex was subjected to single point evaluations in the respective solvent at M062X/6-311++G(d,p)//SDD level of theory. The difference between the gas-phase and SMD energies was used to calculate the solvation free energy, ΔG_solv_^ε^, of each metal complex. The outgoing Ca^2+^ and incoming Ln^3+^ are considered to be in a bulk aqueous environment (ε = 78) outside the binding pocket. Accordingly, their experimentally evaluated/estimated hydration free energies of −359.7 kcal mol^−1^ (for Ca^2+^ [44] and Ln^3+^ (from −751.7 to −849.7 kcal mol^−1^, see Table 4 below), respectively were used in the computations. The experimental hydration free energy of water of −6.3 kcal mol^−1^ [54] was employed as well. The cation exchange free energy in a protein-binding pocket characterized by an effective dielectric constant was evaluated as

∆G^ε^ = ∆G^1^ + ∆G_solv_^ε^ ([Ln^3+^-protein]) − ∆G_solv_^ε^ ([Ca^2+^-protein]) − ∆G_solv_^78^ ([Ln^3+^-aq]) + ∆G_solv_^78^ ([Ca^2+^-aq]) (4)

Note that Equation (4) allows us to separate electronic effects (interactions between the metal cation and ligands lining the binding pocket) from other effects such as the solvent accessibility and the effective dielectric constant of the active center, which are controlled by the protein matrix. 

**Table 4:** Ionic radii, hydration free energies and effective core potentials for lanthanides.

Metal Cation	Ionic Radius ^a^ (Å)	ΔG_hydration_ (kcal mol^−1^) ^b^	ECP
La^3+^	1.03	−751.7	MWB46
Ce^3+^	1.02	−764.8	MWB47
Pr^3+^	0.99	−775.6	MWB48
Nd^3+^	0.983	−783.9	MWB49
Pm^3+^	0.970	−788.7 ^c^	MWB50
Sm^3+^	0.958	−794.7	MWB51
Eu^3+^	0.947	−803.1	MWB52
Gd^3+^	0.938	−806.6	MWB53
Tb^3+^	0.923	−812.6	MWB54
Dy^3+^	0.912	−818.6	MWB55
Ho^3+^	0.901	−827.3 ^c^	MWB56
Er^3+^	0.89	−835.3	MWB57
Tm^3+^	0.88	−840.1	MWB58
Yb^3+^	0.868	−845.8 ^c^	MWB59
Lu^3+^	0.861	−849.7 ^c^	MWB60

^a^ From Greenwood and Earnshaw, 1997 [55]. ^b^ Experimentally determined hydration free energies (from Marcus [44]) unless indicated. ^c^ Estimated from the ΔG_hydration_/Ionic radius correlation (R^2^ = 0.9926); Figure 4.

## 4. Conclusions

The present research offers the first systematic investigation of the ability of the entire series of lanthanides to substitute for Ca^2+^ in biological systems (to the best of our knowledge). The calculations performed reveal that the major determinant of the Ca^2+^/Ln^3+^ selectivity in calcium proteins is the net charge of the calcium binding pocket; the more negative the charge, the higher the competitiveness of the trivalent Ln^3+^ with respect to its Ca^2+^ contender (Figure 2). The solvent exposure of the binding site also influences the process; buried active centers with net charge of −4 or −3 are characterized by higher Ln^3+^ over Ca^2+^ selectivity (Figure 2A,B), whereas it is the opposite for sites with overall charge of −1 (Figure 2C). These findings are in line with our earlier results on Ca^2+^ → La^3+^ competition [35].

Within the series, the competition between La^3+^ and its fellow lanthanides is determined by the balance between two competing effects: electronic (favoring heavier lanthanides) and solvation (generally favoring the lighter lanthanides). The dependency is not straightforward, as ΔΔG^4/29^ fluctuate between positive and negative values, following semi-sinusoidal-like distribution (Figure 3). The calculations demonstrate that lighter lanthanides (especially Ce^3+^, Pr^3+^ and Eu^3+^) are poor competitors to their namesake lanthanum, whereas their counterparts from the middle of the series (Gd^3+^, Tb^3+^ and Dy^3+^) are predicted to have greater affinity to the binding pocket than lanthanum. The dielectric properties of the binding cavity only slightly affect the La^3+^/Ln^3+^ competition, since ΔΔG^4^ and ΔΔG^29^ alternate in relatively narrow limits (Table 1, Table 2 and Table 3).

## Figures and Tables

**Figure 4:**
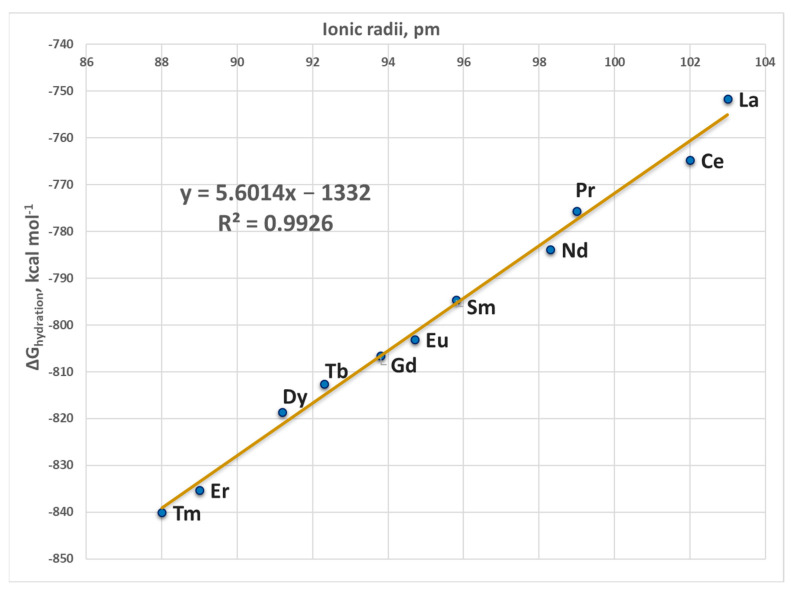
Plot of the experimental ΔG_hydration_ vs. ionic radii for La^3+^/Ln^3+^ cations. There are no experimental data for the hydration free energies of Pm^3+^, Ho^3+^, Yb^3+^ and Lu^3+^.

**Table 1:** Free energies of the Ca^2+^ → Ln^3+^ and La^3+^ → Ln^3+^ exchange (in kcal mol^−1^) for Site 1.

Metal	Ca^2+^ → Ln^3+^			La^3+^ → Ln^3+^		
	ΔG^1^	ΔG^4^	ΔG^29^	ΔΔG^1^	ΔΔG^4^	ΔΔG^29^
La^3+^	−514.6	−47.8	−30.2	0.0	0.0	0.0
Ce^3+^	−524.6	−44.4	−26.7	−10.0	3.4	3.5
Pr^3+^	−532.6	−41.6	−24.0	−18.0	6.2	6.2
Nd^3+^	−541.7	−42.1	−24.5	−26.7	5.7	5.7
Pm^3+^	−549.1	−45.2	−27.6	−34.5	2.6	2.6
Sm^3+^	−556.7	−46.3	−28.8	−41.7	1.5	1.4
Eu^3+^	−563.8	−45.4	−28.0	−49.2	2.4	2.2
Gd^3+^	−570.7	−48.8	−31.4	−56.1	−1.0	−1.2
Tb^3+^	−577.8	−50.1	−32.7	−63.4	−2.3	−2.5
Dy^3+^	−584.6	−50.7	−33.3	−70.0	−2.9	−3.1
Ho^3+^	−591.1	−48.5	−31.2	−76.5	−0.7	−1.0
Er^3+^	−594.9	−44.4	−27.3	−80.3	3.4	2.9
Tm^3+^	−601.3	−46.0	−29.0	−86.7	1.8	1.2
Yb^3+^	−607.8	−46.8	−29.8	−93.2	1.0	0.4
Lu^3+^	−613.0	−48.1	−31.1	−98.4	−0.3	−0.9

**Table 2:** Free energies of the Ca^2+^ → Ln^3+^ and La^3+^ → Ln^3+^ exchange (in kcal mol^−1^) for Site 2.

Metal	Ca^2+^ → Ln^3+^			La^3+^ → Ln^3+^		
	ΔG^1^	ΔG^4^	ΔG^29^	ΔΔG^1^	ΔΔG^4^	ΔΔG^29^
La^3+^	−439.7	−20.4	−14.4	0.0	0.0	0.0
Ce^3+^	−448.7	−16.1	−10.0	−9.0	4.3	4.4
Pr^3+^	−457.3	−13.7	−7.5	−17.6	6.7	6.9
Nd^3+^	−464.9	−13.1	−7.1	−25.2	7.3	7.3
Pm^3+^	−472.7	−15.8	−10.0	−33.0	4.6	4.4
Sm^3+^	−480.4	−17.1	−11.0	−40.7	3.3	3.4
Eu^3+^	−485.5	−14.7	−8.6	−45.8	5.7	5.8
Gd^3+^	−494.9	−19.8	−13.6	−55.2	0.6	0.8
Tb^3+^	−501.7	−20.8	−14.6	−62.0	−0.4	−0.2
Dy^3+^	−509.5	−22.5	−16.4	−69.8	−2.1	−2.0
Ho^3+^	−515.3	−19.6	−13.7	−75.6	0.8	0.7
Er^3+^	−520.9	−16.8	−11.0	−81.2	3.6	3.4
Tm^3+^	−527.3	−19.1	−13.0	−87.6	1.3	1.4
Yb^3+^	−534.1	−19.7	−13.6	−94.4	0.7	0.8
Lu^3+^	−539.9	−22.1	−16.0	−100.2	−1.7	−1.6

**Table 3:** Free energies of the Ca^2+^ → Ln^3+^ and La^3+^ → Ln^3+^ exchange (in kcal mol^−1^) for Site 3.

Metal	Ca^2+^ → Ln^3+^			La^3+^ → Ln^3+^		
	ΔG^1^	ΔG^4^	ΔG^29^	ΔΔG^1^	ΔΔG^4^	ΔΔG^29^
La^3+^	−309.2	18.0	0.1	0.0	0.0	0.0
Ce^3+^	−316.9	21.2	4.6	−7.7	3.2	4.5
Pr^3+^	−327.6	23.3	5.2	−18.4	5.3	5.1
Nd^3+^	−336.4	22.8	4.5	−27.2	4.8	4.4
Pm^3+^	−273.7	19.2	0.8	−35.5	1.2	0.7
Sm^3+^	−349.0	20.6	2.1	−39.8	2.6	2.0
Eu^3+^	−356.1	21.8	3.2	−46.9	3.8	3.1
Gd^3+^	−365.7	15.7	−3.0	−56.5	−2.3	−3.1
Tb^3+^	−373.2	14.1	−4.7	−64.0	−3.9	−4.8
Dy^3+^	−379.6	13.4	−5.5	−70.4	−4.6	−5.6
Ho^3+^	−385.6	16.0	−3.0	−76.4	−2.0	−3.1
Er^3+^	−392.0	17.6	−1.3	−82.8	−0.4	−1.4
Tm^3+^	−396.2	18.1	−0.9	−87.0	0.1	−1.0
Yb^3+^	−403.9	15.9	−3.1	−94.7	−2.1	−3.2
Lu^3+^	−407.8	15.8	−3.2	−98.6	−2.2	−3.3

## Data Availability

The data presented in this study are available on request from the corresponding author.
